# Evaluation of Therapeutic Efficacy and Safety of Tranexamic Acid Local Infiltration in Combination with Topical 4% Hydroquinone Cream Compared to Topical 4% Hydroquinone Cream Alone in Patients with Melasma: A Split-Face Study

**DOI:** 10.1155/2018/8350317

**Published:** 2018-07-02

**Authors:** Zohreh Tehranchinia, Bita Saghi, Hoda Rahimi

**Affiliations:** Skin Research Center, Shahid Beheshti University of Medical Sciences, Tehran, Iran

## Abstract

**Introduction:**

Melasma is an acquired pigmentary disorder characterized by hyperpigmented macules and/or patches affecting sun-exposed skin. Tranexamic acid (TA) can reduce melanin content of epidermis. Thus, we conducted this study to evaluate the efficacy and safety of tranexamic acid local infiltration in combination with topical 4% hydroquinone cream compared to topical 4% hydroquinone cream alone in patients with melasma.

**Material and Methods:**

This study was a prospective assessor- and analyst-blind, randomized split-face clinical trial which was performed on patients with bilateral malar epidermal melasma. A total of 55 patients were enrolled, and each side of their face was randomly allocated to either TA+HQ or HQ alone treatment. The MASI score was applied as an objective measurement to compare two treatment groups. The patient's satisfaction of melasma treatment was evaluated using a four-scale grading, as well.

**Results:**

The mean of MASI score in week 16 decreased in both groups significantly (p < 0.01). The therapeutic outcomes were significantly better in TA+HQ group than HQ group (p=0.001). Patients satisfaction with treatment was significantly higher in the TA + HQ group. The difference between the two groups regarding side effect occurrence was not statistically significant.

**Conclusion:**

Addition of tranexamic acid injections to conventional hydroquinone therapy can increase the efficacy of topical treatment.** This trial is registered with **IRCT2015110324865N1.

## 1. Introduction

Melasma, which may be symmetric or asymmetric, is an acquired pigmentary disorder characterized by hyperpigmented macules and/or patches affecting sun-exposed skin [1–4]. The precise etiology of melasma is unknown, but ultraviolet radiation, pregnancy, oral contraceptives (OCP), hormonal therapy, phototoxic and antiseizure drugs, and thyroid dysfunction are considered risk factors of melasma [1, 2, 4–6]. Melasma may impose major psychological and emotional burden on patients and affect their quality of life. Common therapeutic approaches for melasma include topical hydroquinone (HQ), azelaic acid, steroids, chemical peels, and lasers, most of which could not induce remarkable and constant satisfying outcomes [2, 3, 7].

Tranexamic acid (TA), an inhibitor of plasminogen activation, has been recently used in the treatment of melasma in different investigations [4–11]. The exact mechanism of action of tranexamic acid is still unknown, but the evidence shows that it can reduce the melanin content of epidermis, decrease the dermal vascularity, and mast cell numbers[7].

Although tranexamic acid is currently the only drug which can prevent hormonal, UV-induced, and keratinocyte-derived melanocyte activation and has shown promising results in treatment of melasma, blinded clinical trials that compare this drug with other conventional treatments are few. Thus, we conducted this study to evaluate the efficacy and safety of tranexamic acid local infiltration in combination with topical 4% hydroquinone cream compared to topical 4% hydroquinone cream alone in patients with melasma.

## 2. Patients and Methods

This study was a prospective assessor- and analyst-blind, randomized split-face clinical trial which was performed on patients with bilateral malar epidermal melasma. Exclusion criteria included pregnancy, lactation, any melasma treatment within 1 month prior to study, taking OCP or phototoxic drugs during 1 month prior to study, and history of vitiligo. The study was approved by our ethics committee and was registered on our national clinical trial registry (registration no. IRCT2015110324865N1). Informed consent was obtained from all patients before participation in the study.

A total of 55 patients were enrolled, and each side of their face was randomly allocated to either TA+HQ or HQ alone treatment. All patients applied topical HQ 4% cream (Merck Company, Germany) on his/her both malar sides every night, for 12 weeks. The TA+HQ side of the face received additional 1 mL TA (Caspian Tamin Company, 100 mg/ml) intradermal injection with 1 cm intervals by an insulin syringe with a 30-gauge needle at weeks 0, 4, 8, and 12. All patients were recommended to use a sunscreen cream with sun protection factor of 50.

The pretreatment assessment including comprehensive history, physical examination, melasma and severity index (MASI) scoring, and colour photographs were done by an expert dermatologist. The visit sessions were repeated at weeks 4, 8, 12, and 16, and at each session the new photographs were taken and patients were asked for any probable side effects.

The MASI score was applied as an objective measurement to compare two treatment groups more accurately. According to the MASI, the whole face is divided into four areas: 30% the forehead, 30% right malar (RM), 30% left malar (LM), and 10% chin (C). The grade of melasma severity was determined by 3 parameters: area (A), darkness (D), and homogeneity (H). A is scored from 0 (no involvement) to 6 (90-100% involvement) while D and H are scored from 0 (absent) to 4 (maximum). The MASI is then calculated by the following equation:

(1) =0.3DF+HFAF+0.3DMR+HMRAMR+0.3DML+HMLAML+0.1DC+HCAC9

According to our study design, the MASI score was calculated for each malar area, separately. The MASI score for each side of the face was determined according to the photographs of baseline and week 16 (1 month after the completion of treatment) by a dermatologist who was blind to the treatment.

The patient's satisfaction of melasma treatment was evaluated using a four-scale grading: poor: response rate= 0-25%; fair: response rate= 25-50%; good: response rate=50-75%; excellent: response rate=75-100%.

All collected data were reported as number or frequency percentage for qualitative variables and mean ±standard deviation for quantitative variables. The data analyst was blind to treatment group allocation. Statistical analysis was performed by Statistical Package for the Social Sciences (SPSS 16.0.0, IBM, Chicago-IL, USA). Two sample t-test, paired samples t-test, and analysis of variance (ANOVA) were used to compare parameters. Variables were considered significant for a confidence interval of 95% (p<0.05).

## 3. Results

All 55 included patients completed the study. Baseline demographics and clinical characteristics of the patients are summarized in Table 1.

**Figure 1:**
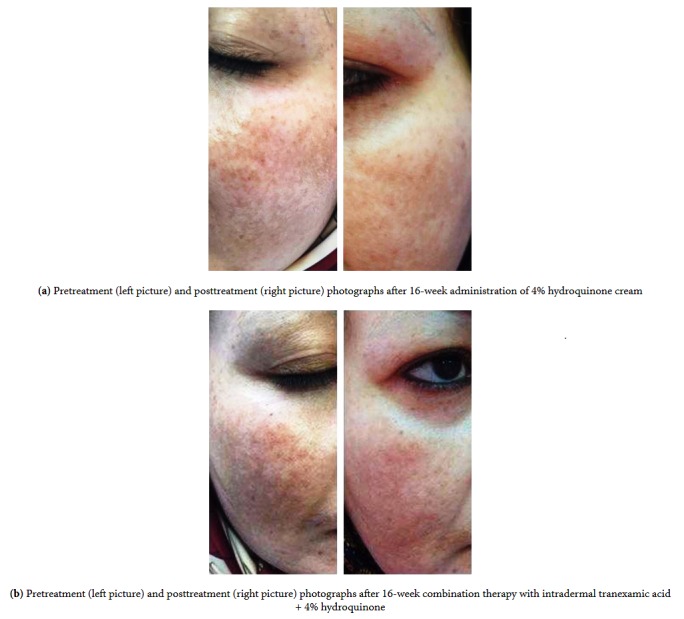


**Figure 2:**
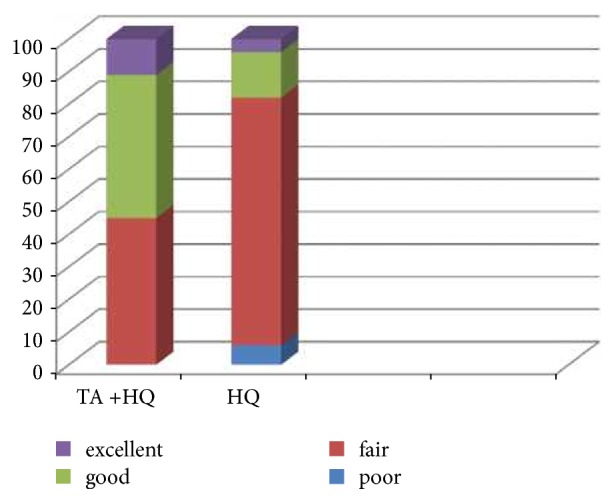
Comparison of patients satisfaction in two groups. HQ; hydroquinone, TA; tranexamic Acid. Poor: response rate = 0-25%; fair: response rate = 25-50%; good: response rate = 50-75%; excellent: response rate = 75-100%.

At baseline, the mean of MASI score was 5.165±1.875 in TA+HQ group 5.204±1.935 in HQ group with no significant difference between two groups (p=0.08). The mean of MASI score in week 16 decreased in both groups significantly (p < 0.01), which showed the efficacy of both therapeutic regimens. In week 16, the mean of MASI score was 1.769±0.981 in TA+HQ group (Figure 1) and 2.926±1.219 in HQ group (Figure 2). The therapeutic outcomes were significantly better in TA+HQ group than HQ group (p=0.001) (Table 2).

Patients satisfaction with treatment was significantly higher in the TA + HQ group with 55% of patients reporting good to excellent response compared to 16% in the HQ group (P < 0.001) (Figure 2).

The side effects in TA+ HQ group were minor and transient including erythema (47.3%) and pruritus at the site of injection (10.9%). The side effects in HQ group were erythema in 50.9% of cases and pruritus in 12.7% of cases. The difference between the two groups regarding side effect occurrence was not statistically significant (P = 0.43).

## 4. Discussion

This study showed that combination therapy with intradermal tranexamic acid and topical hydroquinone was more effective than conventional therapy (hydroquinone) in the treatment of melasma with less side effects.

The mechanism of action of TA in treatment of melasma is not completely understood but it seems to suppress UV-induced plasmin activity in keratinocytes. Tranexamic acid inhibits the binding of plasminogen to keratinocytes, consequently reducing the synthesis of prostaglandins, which are well-known stimulators of tyrosinase activity [12]. Moreover, plasmin increases diffusible forms of vascular endothelial growth factor (VEGF), resulting in angiogenesis. Thus, inhibition of plasmin by TA leads to reduction of angiogenesis.

Several studies have demonstrated the efficacy of TA in treatment of melasma [4–11], but in a large number of these studies, TA have been used orally with numerous systemic side effects such as menstrual irregularities, gastrointestinal symptoms, and orthostatic imbalances [13–15].

Topical administration of TA was assessed in a randomized double blind split-face trial study by Kanechorn Na Ayuthaya et al. They reported that therapeutic effect of 5% TA gel on melasma was neither superior to nor different from its vehicle [16]. Na et al. reported that combination of oral and topical TA reduces epidermal pigmentation of melasma and reverses melasma related alterations in the dermis such as vessels and mast cells number [17]. In another split-face trial, Ebrahimi et al. used topical 3% TA suspension for one side of the face and a 3% hydroquinone, 2% vitamin C, and 0.01% dexamethasone suspension for the other side of the face. A significant decreasing in MASI score was observed in both groups, but with no significant difference between them [1].

There are only 4 trials which have investigated the effect of microneedling or microinjection in treatment of melasma. Lee et al. conducted a prospective open-label study to evaluate the effectiveness of weekly microinjection of TA in treatment of melasma. They observed statistically significant decline in MASI score from baseline to 8 and 12 weeks [18]. In another study, Steiner et al. evaluated the efficacy of intradermal injection of TA versus topical administration of 3% TA cream in treatment of melasma. They reported that both treatments improved melasma with no significant difference [19]. Budamakuntla et al. compared microinjection of TA with its microneedling and found no significant difference in MASI score and patient satisfaction between these two modalities [2].

In a recent study, Elfar et al. conducted a clinical trial to compare the therapeutic effect of intradermal injection of TA, glycolic acid peeling 50%, and topical silymarin cream in treatment of melasma. The participants were followed up for 3 months after the treatment. They reported that the microinjection of TA significantly decreased the MASI score; however, its response rate was less than glycolic acid peeling than silymarin cream [20].

Like most other studies, we found a better response rate with combination of TA and HQ. However, the limitation of our study was the short follow-up period of only 1 month. As recurrence of melasma is common in spite of appropriate treatment, studies with long-term follow-up are recommended.

## 5. Conclusion

In conclusion, addition of tranexamic acid injections to conventional hydroquinone therapy can increase the efficacy of topical treatment. In our study, major and statistically significant improvement in MASI scores was achieved by combining these two treatment regimens with fewer side effects. Therefore, it may be helpful in treatment of all cases of melasma, especially the refractory ones.

## Figures and Tables

**Table 1:** Baseline demographics and clinical characteristics of patients with melisma.

**Gender**, No. (%)	
Female	49 (89.1)
Male	6 (10.9)

**Age**, years	
Mean± SD	35.93±5.9
(Range)	(27-51)

**Duration of disease **, years	
Mean± SD	6.24±2.7

**Fitzpatrick's skin type**; N (%)	
2	9 (16.4)
3	35 (63.3)
4	11 (20)

**Family history; **N (%)	
Positive	28 (50.9)
Negative	27 (49.1)

**History of oral contraceptive intake; **N (%)	
Positive	30 (54.5)
Negative	25 (45.5)

**Table 2:** MASI score before and after treatment in two groups.

	**Tranexamic acid +**	**Hydroquinone**	**p-value**
	**Hydroquinone**		
**Baseline MASI Score**	5.165±1.875	5.204±1.935	0.08
**(Mean**±**SD)**			
**MASI Score in week 16**	1.769±0.981	2.926±1.219	0.001
**Mean**±**SD**			

## Data Availability

The data used to support the findings of this study are available from the corresponding author upon request.
